# Modification of Concrete Composition Doped by Sewage Sludge Fly Ash and Its Effect on Compressive Strength

**DOI:** 10.3390/ma16114043

**Published:** 2023-05-29

**Authors:** Tomasz Kalak, Patrycja Szypura, Ryszard Cierpiszewski, Malgorzata Ulewicz

**Affiliations:** 1 Department of Industrial Products and Packaging Quality, Institute of Quality Science, Poznan University of Economics and Business, Niepodleglosci 10, 61-875 Poznan, Polandryszard.cierpiszewski@ue.poznan.pl (R.C.); 2 Faculty of Civil Engineering, Czestochowa University of Technology, Akademicka 3 Street, 42-200 Czestochowa, Poland

**Keywords:** concrete, compressive strength, sewage sludge fly ash, destructive testing

## Abstract

The sustainable development of construction materials is an essential aspect of current worldwide trends. Reusing post-production waste in the building industry has numerous positive effects on the environment. Since concrete is one of the materials that people manufacture and use the most, it will continue to be an integral element of the surrounding reality. In this study, the relationship between the individual components and parameters of concrete and its compressive strength properties was assessed. In the experimental works, concrete mixes with different contents of sand, gravel, Portland cement CEM II/B-S 42.5 N, water, superplasticizer, air-entraining admixture, and fly ash from the thermal conversion of municipal sewage sludge (SSFA) were designed. According to legal requirements in the European Union, SSFA waste from the sewage sludge incineration process in a fluidized bed furnace should not be stored in landfills but processed in various ways. Unfortunately, its generated amounts are too large, so new management technologies should be sought. During the experimental work, the compressive strength of concrete samples of various classes, namely, C8/10, C12/15, C16/20, C20/25, C25/30, C30/37, and C35/45, were measured. The higher-class concrete samples that were used, the greater the compressive strength obtained, ranging from 13.7 to 55.2 MPa. A correlation analysis was carried out between the mechanical strength of waste-modified concretes and the composition of concrete mixes (the amount of sand and gravel, cement, and FA), as well as the water-to-cement ratio and the sand point. No negative effect of the addition of SSFA on the strength of concrete samples was demonstrated, which translates into economic and environmental benefits.

## 1. Introduction

Construction-related advancements necessitate the ongoing development of building materials, particularly concrete. The competition between various construction materials is intensifying as globalization advances. In order to find and create products with the best strength and durability, there is therefore a lot of research being conducted in the area of concrete durability. Concrete is used in the construction of buildings because it is strong, durable, and has superior strength characteristics. Sand, gravel, and cement are just a few of the ingredients used to make concrete. In order to impart particular performance attributes, extra chemical admixtures are also used in the concrete mix [1]. The United States (72 million metric tons (MMT)), the Netherlands (54 MMT), Turkey (12 MMT), India (12 MMT), France (11 MMT), Italy (10 MMT), Bulgaria (8.4 MMT), Spain (5.7 MMT), and Poland (5.5 MMT) were the world’s top producers of sand and gravel in 2021 [2]. For the past ten years, 4.1 billion metric tons of cement have been produced worldwide [3]. China (2100 MMT), India (370 MMT), Vietnam (120 MMT), the United States (95 MMT), and Turkey (85 MMT) were the principal countries producing the most cement in the world in 2022 [4]. By-products from the cement-making process, namely, CO_2_, NO_x_, and SO_2_, are emitted into the atmosphere. China, which released 853 Mt of CO_2_ into the atmosphere in 2021, is the world leader in CO_2_ emissions into the atmosphere from cement production. India (149 MMT), Vietnam (54.1 MMT), Turkey (44.4 MMT), the United States (41.2 MMT), Saudi Arabia (28.7 MMT), Indonesia (28.6 MMT), Japan (23.8 MMT), South Korea (23.7 MMT), and Iran (22.5 MMT) are the countries that come behind it [5]. Different industrial by-products are utilized in concrete as partial substitutes for cement to lower CO_2_ emissions. Thanks to this, the landfilling of waste is reduced, which becomes a new raw material used by the construction industry in accordance with the principles of sustainable development and the circular economy [6,7,8,9,10].

Fly ash (FA) and waste glass sludge are two of the industrial wastes that are most commonly utilized to produce concrete. Landfilling the waste causes significant environmental issues, such as the tainting of ground or surface waters, an increase in soil alkalinity, and detrimental impacts on vegetation and other living organisms. Thus, using waste materials has environmental benefits by reducing landfilling and lowering CO_2_ emissions into the atmosphere [1,11,12]. Furthermore, waste materials are added to increase mechanical qualities such as compressive and tensile strength [13].

Fly ash (FA), a by-product of combustion operations, is one of the useful waste materials added to concrete mixtures. The fine-grained dust, which is made up of grains of erratic sizes and shapes, is a useful mineral additive. FA increases the workability of concrete mixes, but it also makes concrete more resistant to the aggressive sulfate environment, slows down the hardening process, makes concrete more resistant to high temperatures (up to 600 °C), reduces frost resistance, reduces shrinkage, and makes hardened concrete stronger and more durable. The adroit application of FA’s features results in concrete with improved properties that are suited to receivers’ requirements. An additional benefit is the reduced price of concrete compared to that made using only cement. A further advantage is that the concrete costs are less than those of concrete produced only with cement. Numerous researchers have discussed in the literature the impact of adding different forms of FA on the characteristics of concrete [14,15,16,17,18,19]. FA waste from the burning of hard coal [14,15], biomass [17,19,20], and sewage sludge from municipal facilities [18,21,22,23] was used to modify mortars and concrete. Concrete [22,23] and cement [24] were enhanced with dried sewage sludge. Dried sewage sludge was added to concrete in levels ranging from 50 to 15% as a substitute for cement. It has been demonstrated that the replacement of cement with FA in concrete increased the compressive strength after 28 days of curing, but the attained strength of waste-modified concrete was less than that of control samples [22]. These wastes were also substituted for sand in concrete from 2.5 up to 12.5%. According to reports, replacing sand with waste up to 7.5% does not affect the characteristics of concrete’s strength [23]. Fly ash from sewage sludge (SSFA) in the amount of 15 to 30% can be added to cement mortars without significantly changing their mechanical properties [25]. The heat of hydration in cement mortars is affected by adding SSFA in amounts ranging from 2.5 to 20%. Along with an increase in the exchange of cement with waste, the rate of heat release was slowed down, especially during the early stages of mortar binding [26]. In this study, SSFA waste was added to concrete mixtures, which has positive social and economic effects. Fly ash is produced from the incineration of sewage sludge from local municipalities. The combustion technology is a modern and advanced solution for the thermal treatment of sewage sludge. Low emissions of pollutants, high energy and combustion efficiency, the ability to recover energy, compatibility with a variety of fuels, and simplicity of the installation operation are only a few of the benefits. This kind of waste production is steadily rising everywhere, including Poland. According to statistics, this country produced 1048.7 thousand tons of sewage sludge in 2019; only 159.7 thousand of those tons were thermally converted, and roughly 113.3 thousand tons were kept in landfills [27,28]. Since it is difficult to effectively use such a large amount of sewage sludge in land reclamation or other applications, the share of thermal treatment methods will rise. As a result, there will be an increase in the amount of SSFA created, which requires the development of effective management techniques.

Numerous financial and environmental advantages come from adding SSFA to cement and concrete. These include removing sewage sludge to free up space in landfills that already exist, cutting costs and raising the quality of many building materials, lowering waste disposal costs, including those associated with landfilling, lowering the use of a significant amount of primary raw materials, implementing a sustainable development economy system by turning sewage sludge waste into new useful products, conserving energy, and lowering the emissions of NO_x_, CO_2_, and other pollutants. Public education or a greater understanding of the advantages of waste recycling may be an additional benefit. The usage of the SSFA additive has benefits as well as drawbacks, such as its decreased pozzolanic activity, which results in a reduction in strength qualities and an increase in water consumption, although these drawbacks can be offset by grinding, appropriate modification, and treatment. Another limitation is the lower reactivity of these materials compared to cement flour [28,29,30,31,32,33,34,35].

There are reports of laboratory attempts to utilize SSFA waste for use in regular concrete and cement mortars in the literature. However, the findings of this research are unclear and do not suggest that they might be implemented in the manufacturing of regular concrete products. Therefore, a study was undertaken to determine compressive strength, one of the most crucial parameters for modified concrete. SSFA may be used as an addition in the creation of concrete mixes in line with the applicable Polish legislation (Journal of Laws of 2016, item 108) [36] and the Directive of the European Parliament and of the Council (EU/2010/75) [37]. The SSFA produced using circulating fluidized bed combustion (CFBC) technology should be considered a possible material for use in the manufacturing of building materials, given the significance of zero-emissions, sustainability, and the circular economy [38,39,40,41,42].

The aim of the research was to determine the influence of SSFA waste generated during the combustion process of municipal sewage sludge in a fluidized bed furnace on the compressive strength of different classes of concrete. The correlation of the mechanical strength of waste-modified concretes with respect to the composition of concrete mixes (amount of sand and gravel, cement, SSFA), water-to-cement ratio, and sand point was analyzed.

The use of fly ash as a waste product from the incineration of local municipal sewage sludge utilizing modern circulating fluidized bed combustion (CFBC) technology is the scientific novelty of the research. Using this technology to thermally treat sewage sludge has a number of advantages, including a sizable decrease in waste weight, the recovery of thermal energy during combustion, high process efficiency, low pollutant emissions, the reduction of odors, system stability, and low susceptibility to changes in sludge composition. SSFA may be added to concrete, bricks, asphalt, ceramics, or other materials in addition to serving as a filter medium. It has a high content of mineral substances, and its quality and composition depend on the chemical composition of the sludge, the conditions of the combustion process, flue gas cleaning, and the cooling rate. According to the new regulations in the European Union, landfilling of waste should be avoided, and methods of safe and ecological disposal are to be sought. In Poland, the problem is the excessive amount of sewage sludge produced; hence, more and more thermal processing installations are being built. These activities are in line with the trends of sustainable development and the circular economy. Hence, this research work is a response to regional problems in Poland related to waste management and brings suggestions that may be a partial solution to these problems. In Central Europe, the construction industry is a booming sector that can partially absorb excess SSFA and convert it into new building materials.

The present studies in this manuscript are limited to the compressive strength of concrete and the analysis of correlations with individual components. The limitation also applies to a period of up to 28 days. Because compressive strength is a feature that serves one of the most crucial roles in concrete structures, tensile and flexural strength tests were left out. Because the experiments were concentrated on the strength characteristics of the concrete samples, other tests, such as durability evaluations, were not conducted.

## 2. Experimental Procedure

### 2.1. SEM-EDS Analysis

The elemental composition of SSFA samples was examined with a scanning electron microscope (SEM) Hitachi S-3700N (accuracy: <0.4 nm for all fields of view and pixel sizes; manufacturer: Hitachi High-Technologies Corporation, Tokyo, Japan) with an attached Noran SIX energy dispersive X-ray spectrometer (EDS) microanalyzer (ultra-dry silicon drift type with resolution (FWHM) 129 eV, accelerating voltage: 20.0 kV; manufacturer: Thermo Electron Corporation, Schönwalde-Glien, Germany).

### 2.2. Transmission Electron Microscopy (TEM)

High-resolution transmission electron microscopy (HRTEM) and scanning transmission electron microscopy (STEM) were carried out using a JEOLARM 200F (accuracy: the highest point resolution is around 0.05 nm). SSFA samples for analysis using transmission electron microscopy were prepared by deposition of catalysts onto holey carbon films supported by a copper TEM grid.

### 2.3. XRD Analysis

X-ray diffraction measurements were made using the D8 Advance diffractometer (apparatus accuracy is approximately 100% for phase identification and 86% for three-step-phase-fraction quantification; manufacturer: Bruker AXS Advanced X-ray Solutions Gmbh, Karlsruhe, Germany). In configuration, the diffractometer is equipped with a Johansson monochromator (λ_Cu Kα1_ = 1.5406 Å) and a LynxEye silicon strip detector. The minimum measurement angle is 0.6° 2Θ deg. The XRD powder diffraction method requires the delivered sample to be carefully powderized. A standard measuring dish has a container for powder with a diameter of 25 mm and a depth of 1.5 mm. Before measurement, the sample powder needs to be lightly pressed.

### 2.4. Preparation of Concrete Mixes for Tests

Samples for testing the compressive strength were taken in accordance with the PN-EN 12390-3:2019-07 standard [43]. The fresh concrete mixes (tested recipes C8/10, C12/15, C16/20, C20/25, C25/30, C30/37, and C35/45), produced at the concrete mixing plant, were taken into a container whose walls were previously lubricated with agents, preventing the concrete mixture from sticking to the container. The concrete mixture was transferred from the container into a mold with dimensions of 150 mm × 150 mm × 150 mm. Two layers of the mixture, each no taller than 20 cm, were vibrated throughout the molding process. The amount of compaction of the concrete mix in the mold and venting determine how long the concrete mix is vibrated for. It was taken care not to shake the mixture for an extended period of time, as this could cause ingredient segregation or the laitance effect of the cement to show on the surface (bleeding). The sample’s surface was vibrated and then lapped to level it.

In the next stage, the samples were then completely immersed in water at 20 °C for a minimum of 24 h for maturation in molds. Then, before placing them in the testing machine, the samples were dried by wiping excess water from the surface. They were cured in a construction laboratory for 28 days. On the 28th day, the samples were subjected to a compressive strength test using a strength press. All procedures were performed in accordance with the European standards SIST EN 12390-1:2021, SIST EN 12390-2:2019, SIST EN 12390-3:2019, and SIST EN 12390-4:2019 [44,45,46].

The samples’ weights and side length measurements were used to calculate their densities. The testing device’s bearing surfaces as well as the surfaces of the concrete samples were thoroughly cleaned and dried. The sample was set up in the press so that the compressive forces were perpendicular to the direction of sample formation.

### 2.5. Compressive Strength Test

Compressive strength tests were conducted using an Ele International Automatic Compression Machine (accuracy: it was calibrated to within 0.5% of reading, from 1% to 100% of machine capacity; manufacturer: ELE International, Carter Lanes, Klin Farm, Milton Keynes, United Kingdom) that meets the requirements of the PN-EN 12390-4:2020-03 standard [47]. The sample was loaded, and the rate at which the clamping force increased was between 0.2 and 1.0 MPa/s. The apparatus recorded the value of the force with which it acted on the sample’s surface after achieving the sample’s maximum strength value. The compressive strength was calculated using the following formula:

(1) $$CS=\frac{F}{A}$$

where *CS* is the compressive strength (MPa), *F* is the maximum force at the point of failure (N), and *A* is the cross-sectional surface area (m^2^).

The correctness of the test performance was determined using the schemes of correct and incorrect destruction of samples defined in the European Standard SIST EN 12390-2:2009 [44].

## 3. Results and Discussion

### 3.1. Characterization of the Concrete Materials

The investigation was conducted using seven different types of recipes for concrete mixtures of various strengths. Table 1 lists the characteristics and compositions of the concrete mixtures utilized in the study. The percentage of grain masses in the aggregate with a diameter of up to 2 mm is known as the sand point. The w/c ratio is used to determine the correct amount of water and cement in the concrete mixture. It is important to ensure appropriate properties of the mixture (consistency and workability) and functional properties of the concrete (strength, water absorption, and tightness).

The recipe number is the designation of the concrete composition, which determines the class of concrete and is characterized by a certain strength. Each digit in the recipe number has a specific meaning:

- The first digit indicates the type of cement; in this case, each recipe starts with 5, which means that Portland slag cement CEM II/B-S 42.5 N was used. Requirements of the cement are as follows: SO_3_ content ≤ 3.5%, Cl content ≤ 0.1%, initial setting time ≥ 60 min, change in volume ≤ 10 mm, compressive strength after 2 days ≥ 10 MPa, compressive strength after 28 days between 42.5 and 62.5 MPa, Na_2_O_eq_ content ≤ 0.8%.
- The next two digits indicate the class of concrete, e.g., 10 means class C8/10.
- The digit 4 repeated in each of the concrete recipes indicates that aggregate with grain sizes up to 16 mm was used for research.
- The last two digits are successive numbers assigned as a result of creating a recipe.
- The following ingredients were used to make samples of concrete mixes:
  ○ Sand, 0/2 mm (from the Prusim sand mine, Poland);
  ○ Gravel, 2/8 mm and 8/16 mm (from the KSM Rakowice—Górażdże Kruszywa mine, Poland);
  ○ CEM II/B-S 42.5 N cement (from the Górażdże Cement, Poland), symbol designation: CEM II—multi-component Portland cement, 42.5—compressive strength class after 28 days determined in accordance with the Polish standard PN-EN 196-1, S—granulated blast furnace slag, N—normal early strength cement;
  ○ Water from municipal water supplies;
  ○ Fly ash waste produced as a by-product of the combustion of municipal sewage sludge;
  ○ Sikament 400/30 (Sika Poland Sp. zo.o., Warsaw, Poland) plasticizer;
  ○ LPS A94 (Sika Poland Sp. zo.o.) air-entraining admixture.

The physico-chemical properties of 0/2 sand and 2/8 and 8/16 gravel are shown in Table 2 and Table 3.

The chemical composition of CEM II/B-S 42.5 N was determined using the X-ray Diffraction (XRD) method, and the results are presented in Table 4. The physical properties of the material are as follows: specific surface area 4589 cm^2^/g, specific gravity 2.91 kg/dm^3^, initial setting time 240 min, 2-day compressive strength 22.3 MPa, 28-day compressive strength 56.4 MPa.

Sikament 400/30 superplasticizer is a homogeneous dark brown liquid consisting of sulfonated and non-sulfonated polycondensates of sodium and magnesium salts and carbohydrates (naphthalene, lignosulfonate, and gluconate). Sikament 400/30 has the following characteristics: density of 1.1 kg/m3, pH of 5.0 ± 1.0, chloride ion content ≤0.1%, and sodium oxide equivalent ≤4.0%. The superplasticizer admixture’s activity in concrete mixtures is based on the electrostatic repulsion principle. This enables the concrete mix and concrete to have the following properties: intensive wetting of the cement grains by forming a double ionic layer; a significant reduction in friction force; the ability to reduce the amount of mixing water or to liquefy the mix with a constant amount of mixing water; an improvement in workability; the facilitation of the achievement of high water tightness, frost resistance, and reduced water absorption; longer consistency retention; and high durability.

The Sika^®^ Luftporenbildner LPS A-94 air-entraining admixture (Sika Poland Sp. zo.o., Warsaw, Poland) is a homogeneous dark brown liquid consisting of modified tensides. The properties are the following: density of 1 kg/dm^3^, pH of 7.0 ± 1.0, chloride ion content ≤0.1%, and sodium oxide equivalent ≤0.6%. The process by which the additive in concrete mixes works is based on the production of extremely small air bubbles in the cement grout. Correct pore size and arrangement, increased workability due to air entrainment, and high levels of frost resistance in water and salt solutions are all made possible by this process.

Fly ash, used in the studies, was obtained as a result of the incineration of municipal sewage sludge (SSFA) using circulating fluidized bed combustion (CFBC) technology. This technology uses a recirculating loop, i.e., air is blown through a bed of sand at a speed sufficient to suspend particles on the air stream (approx. 1–3 m/s^2^) at a temperature of approx. 800–900 °C. Up to 95% of pollutants can be blocked from entering the atmosphere thanks to this solution. Grain fractions are reported in Table 5 following the granulation analysis. SSFA is a mixture of irregularly shaped and sized particles that frequently form different agglomerates. An examination of the bulk density revealed that it was 0.81 ± 0.02 g/cm^3^. On a vibrating table, the fly ash was further compressed to a density of 1.52 ± 0.03 g/cm^3^. The outcomes of these investigations are crucial for the potential addition of fly ash to building materials. SEM-EDS analysis revealed that it was primarily comprised of SiO_2_, Al_2_O_3_, P_2_O_5_, CaO, CO_2_, Fe_2_O_3_, K_2_O, and other substances (Table 6). The fact that the multi-component SSFA mixture was not a homogeneous product needs to be underlined here. Depending on where in the silo tank the samples were gathered, there may have been a small difference in the quantitative composition of the samples taken. The mixture was nevertheless expected to exhibit the same crystalline phases according to X-ray diffraction analysis. Similar results of SSFA composition analysis can be found in the literature [48,49,50].

The literature reports that the amount of CaO in the mixture affects the setting time. Fly ash is appropriate for concrete mixes when the CaO level is between 5% and 15%, according to Diaz et al. [51]. It was reported that setting time decreases as CaO content rises. The SSFA used in this study showed a CaO content of 10.2%. Measurements were made pointwise on a specific SSFA sample during the analysis using the SEM-EDS method. However, because the substance is a heterogeneous mixture, the oxide content may differ slightly depending on where a measurement site is located on the sample. Calculations utilizing Formula (2) can be used to predict the final setting time of concrete mixtures [52].

(2) $$ST=4838.7\cdot e^{-0.262\cdot \mathrm{CaO}}$$

where *ST* is setting time (min), *e* is exponential number, and CaO is CaO content in fly ash (%, m/m).

According to the formula, the predicted setting time of a concrete mix with a CaO content of 10.2% in SSFA is 333.4 min.

**Figure 1:**
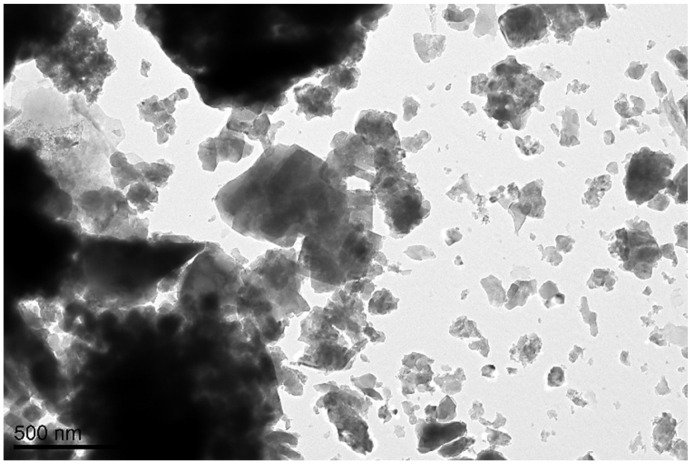
TEM image of SSFA (scale bar: 500 nm).

Figure 1 displays the TEM image of the ash sample. The particles have a flocculent nature, uneven forms, and various diameters. It is possible to see shades of varying intensities; lighter zones correspond to material layers that are thinner, and darker zones correspond to layers that are thicker. Other authors in the literature have also found similar findings for ash TEM images [53,54,55]. The temperature of combustion, the air circulation process, and the cooling rate all impact the morphology of SSFA particles. Because mineral substances were not partially melted in a fluidized bed boiler at temperatures between 800 and 900 °C, irregular shapes, rough structures, or occasionally sharp edges may be the outcome. On the other hand, the enormous surface area created by the SSFA particles’ rough and loose texture may have a negative impact on the amount of water required when employed as an additive in concrete compositions [56,57,58,59].

### 3.2. Analysis of Strength Tests

**Figure 2:**
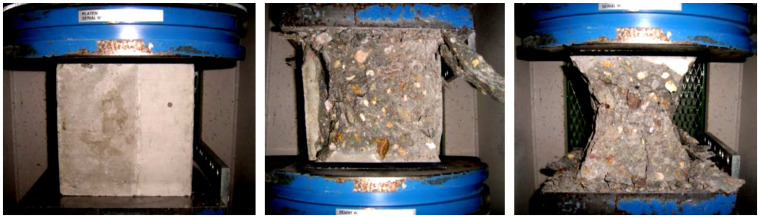
Stages of destruction of the concrete sample during the compressive strength test.

Compressive strength was measured on six cubic samples: C8/10, C12/15, C16/20, C20/25, C25/30, C30/37, and C35/45. The compressive strength of concrete is a parameter determining its durability, and the appropriate selection of its class allows it to be used in a building structure exposed to specific external influences. All test results, including sample weight, density, destructive force at maximum sample load, and compressive strength (Figure 2), are presented in Tables S1–S7 (Supplementary Materials). Table 7 provides a summary of all test results. A destructive force that should not be less than the guaranteed value is also given for each class of concrete. Because of the relationship, as the destructive force is reduced, the concrete’s strength is likewise reduced. There is a particular minimum strength value that each kind of concrete should meet. It is important that the concrete achieves the guaranteed strength without significantly exceeding it, as this could indicate an inadequate composition of the concrete mix [1,60].

According to the results in Table 7, variable proportions of concrete ingredients, including sand, gravel, cement, water, SSFA, and chemical admixtures, have a considerable impact on the strength of concrete mixes. It is clear that the majority of the volume of concrete is made up of the aggregate used to create it, which has a big impact on the properties of both freshly mixed concrete and hardened concrete. Sand serves as a fine aggregate, and when utilized for weaker concretes of the C8/10 and C12/15 classes vs. concretes with enhanced strength of the C20/25–C35/45 classes, a substantial quantitative difference was seen. Due to the fact that the quantitative selection of specific fractions in 2/8 and 8/16 gravel depended on the criteria that the concrete was to fulfill, such as the intended use of environmental conditions or workability, there was no dependence on the class of concrete in this situation. In the construction industry, the aggregate-to-cement ratio is often chosen based on the experience of the production plant or from graphs and tables created using data from laboratory tests. The primary fluidizing agents utilized in the creation of medium- and high-strength concretes are known as superplasticizers. These raw materials used in the research enabled up to a 40% reduction in the amount of mixing water while retaining consistency. Superplasticizers improve workability, increase the strength and durability of concrete, and improve the aesthetics of the surface.

**Figure 3:**
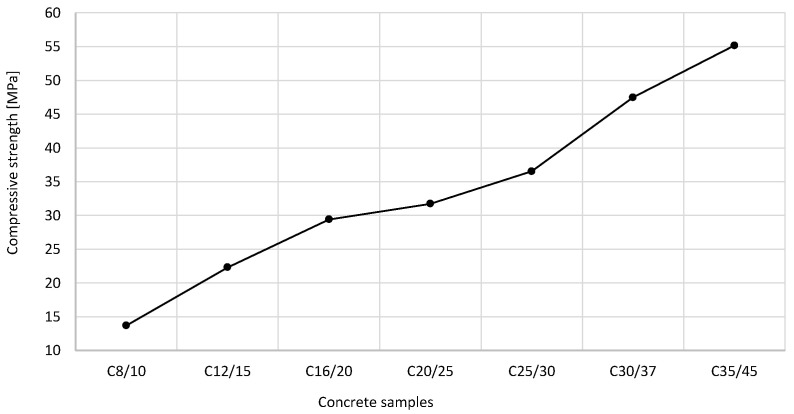
The impact of concrete class sample on compressive strength.

**Figure 4:**
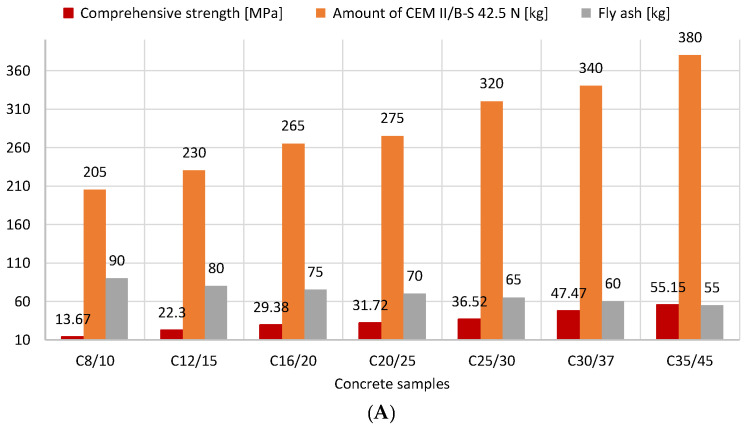

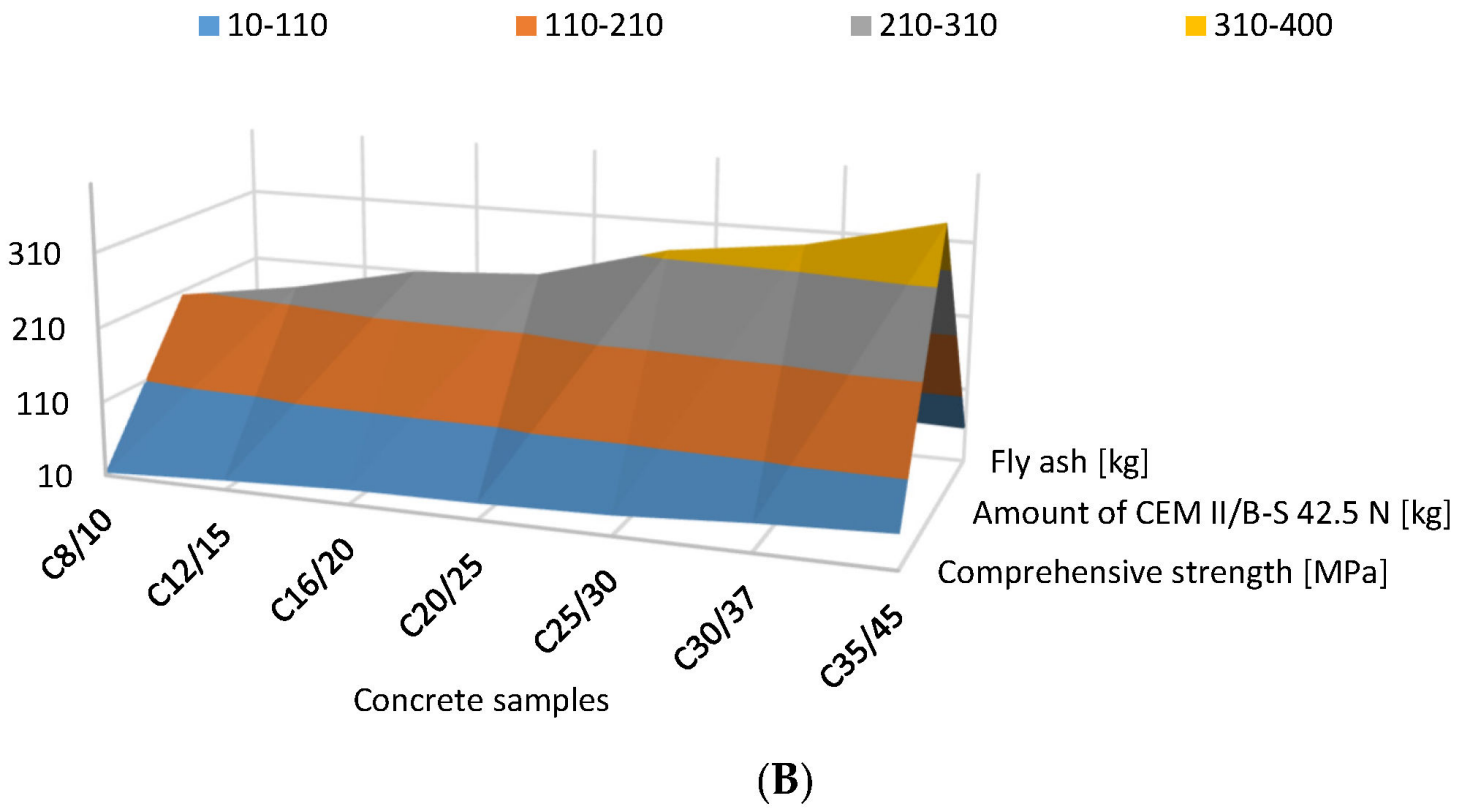
The relationship between the amount of CEM II/B-S 42.5 N cement and SSFA and the compressive strength of concrete mixes (version 2D: (**A**), 3D: (**B**)).

The relationship between the class of concrete samples and their compressive strength is shown in Figure 3. These findings support the hypothesis that the compressive strength of concrete improves as the concrete class increases, namely, from 13.7 MPa (C8/10) to 55.2 MPa (C35/45). The amount of added CEM II/B-S 42.5 N cement is inextricably linked with the compressive strength; it depends on the concrete class and its demand for 1 m^3^ of concrete; however, it should not exceed 400 kg. The cement content proves the durability of concrete to the extent that it affects the water-to-cement (w/c) ratio. The relationship between the amount of cement and SSFA used and the compressive strength of concrete is presented in Figure 4.

It demonstrates that the correct amount of cement should be used to produce the right class of concrete in order to achieve a strength greater than the one that is guaranteed. According to these experiments, increasing the cement addition led to an increase in compressive strength. The addition of SSFA did not weaken the strength properties of concrete, so it can be successfully used. Other researchers who conducted research on the addition of FA published findings that were comparable [16,17,18,19].

**Figure 5:**
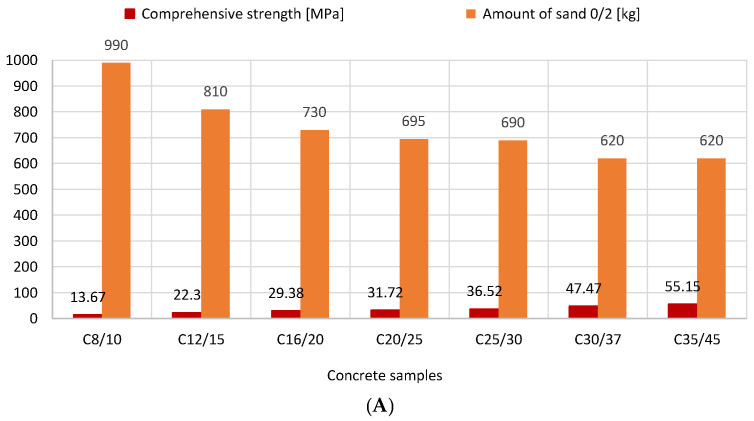

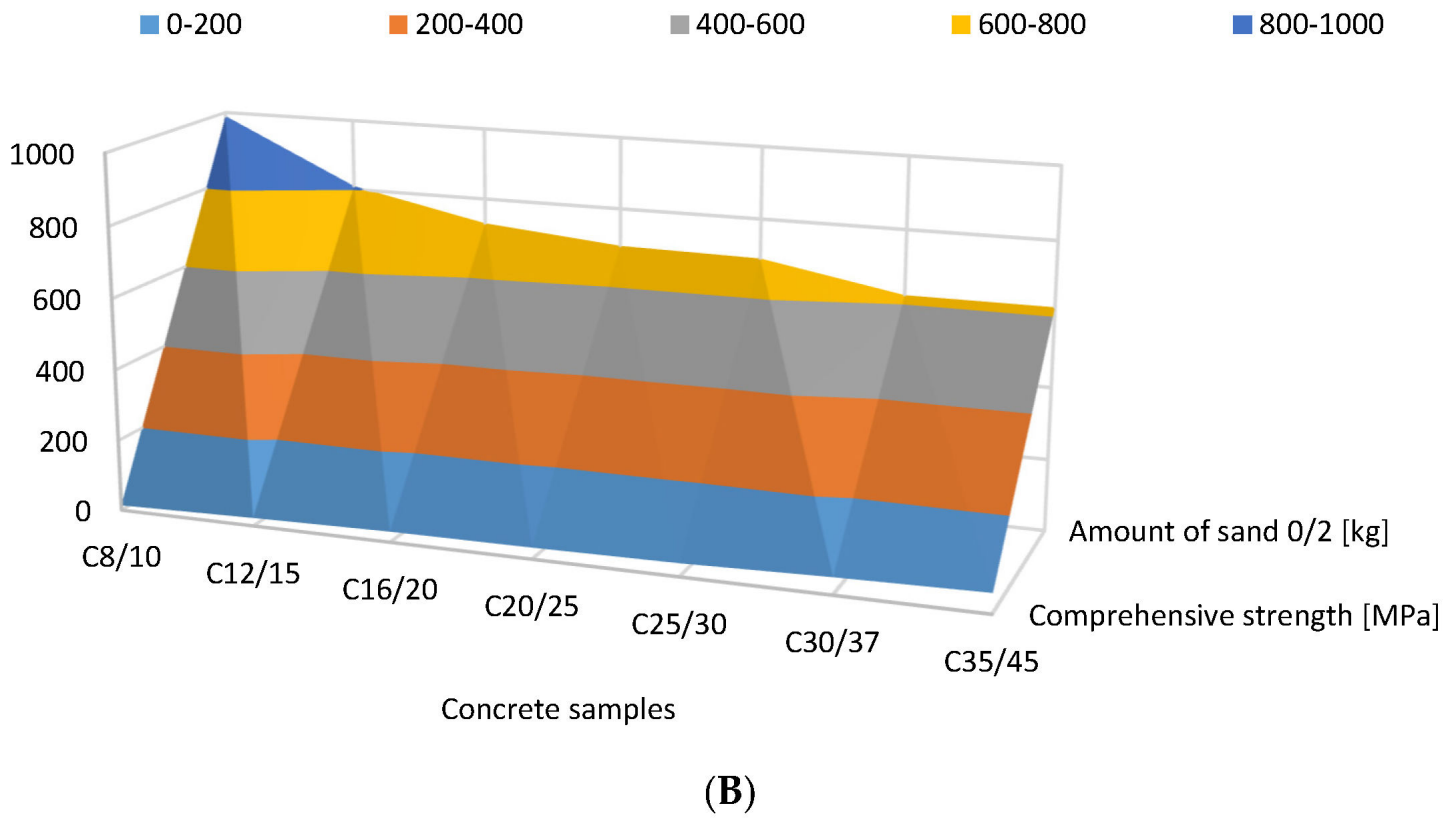
Relationship between amount of 0/2 mm sand and compressive strength of concrete (version 2D: (**A**), 3D: (**B**)).

**Figure 6:**
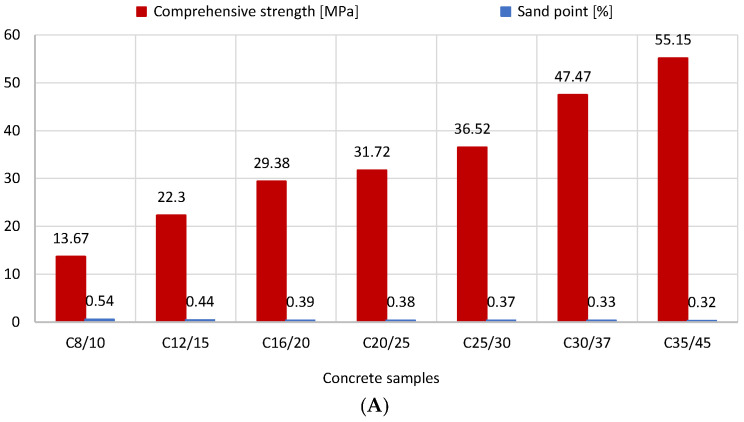

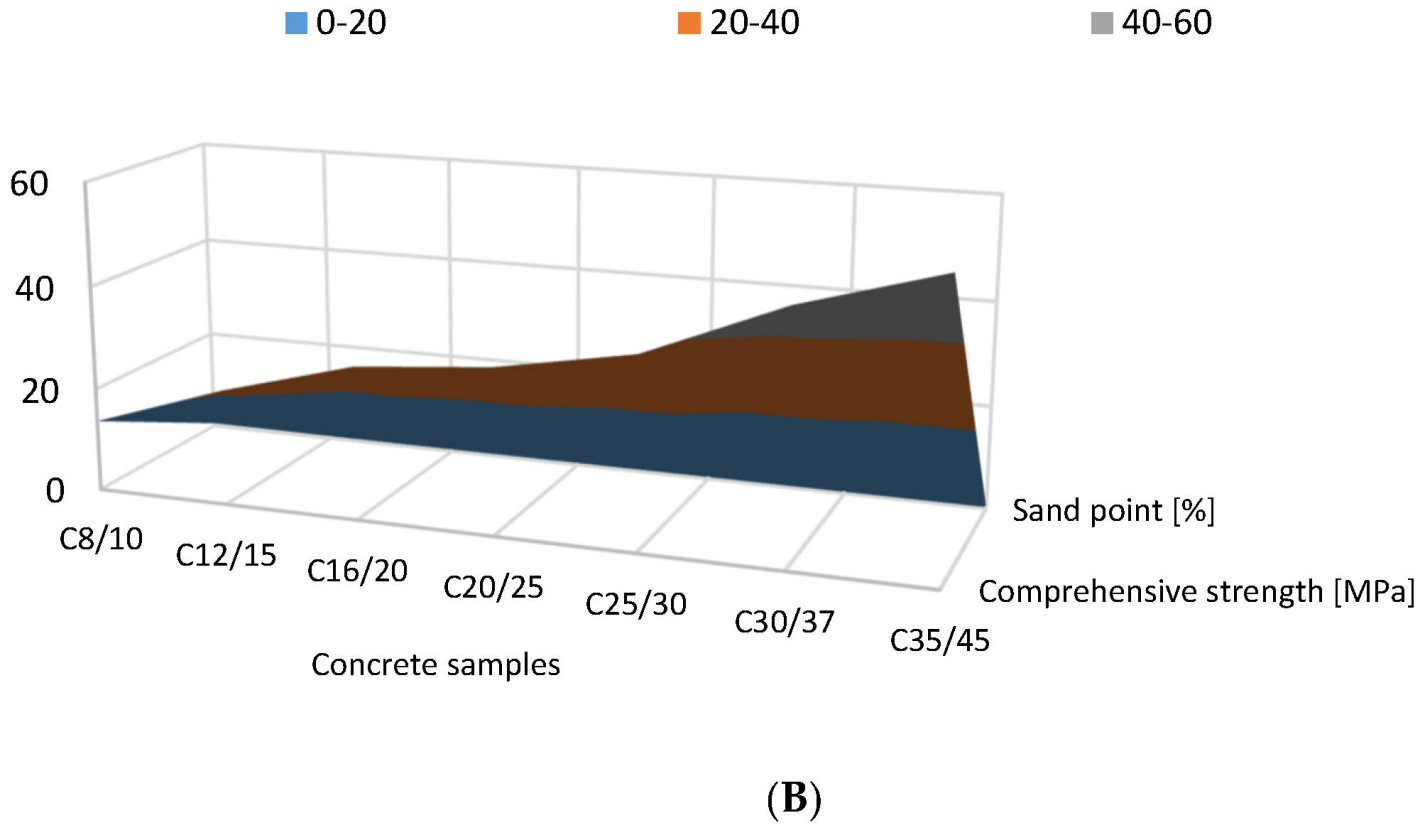
Relationship between sand point and compressive strength of concrete (version 2D: (**A**), 3D: (**B**)).

**Figure 7:**
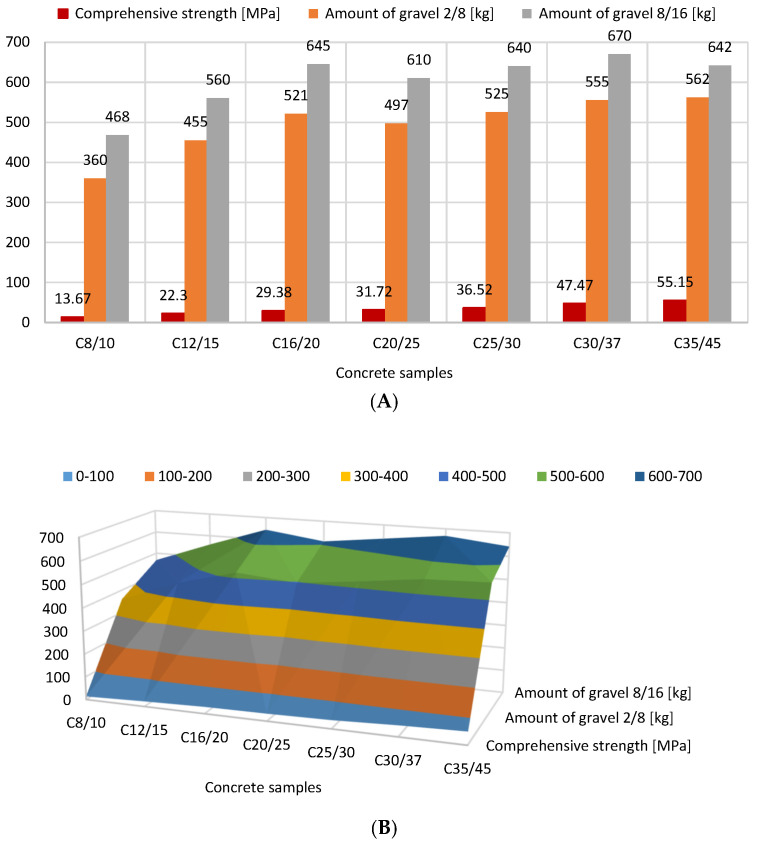
Relationship between amount of 2/8 and 8/16 mm gravel and compressive strength of concretes (version 2D: (**A**), 3D: (**B**)).

Figure 5 illustrates how the amount of sand in concrete mixtures affects compressive strength. Sand was shown to improve compressive strength in concrete mixes from 13.7 MPa (990 kg of sand) to 55.2 MPa (620 kg of sand). The link between the concrete’s compressive strength and the sand point is depicted in Figure 6. The correct sand point for the aggregate should be chosen in order to ensure proper placement and compaction of concrete mixtures while accounting for transport. It can be said that as the concrete strength class increases, so does the water content in the aggregate, which results in a drop in the percentage of the fine aggregate fraction (0–2 mm). The decrease in the sand point, whose defined value influences the strength of the concrete, makes this advantageous. For the addition of 2/8 and 8/16 gravel, however, an inverse relationship was observed. The compressive strength reported increased as more of these ores were introduced (Figure 7).

**Figure 8:**
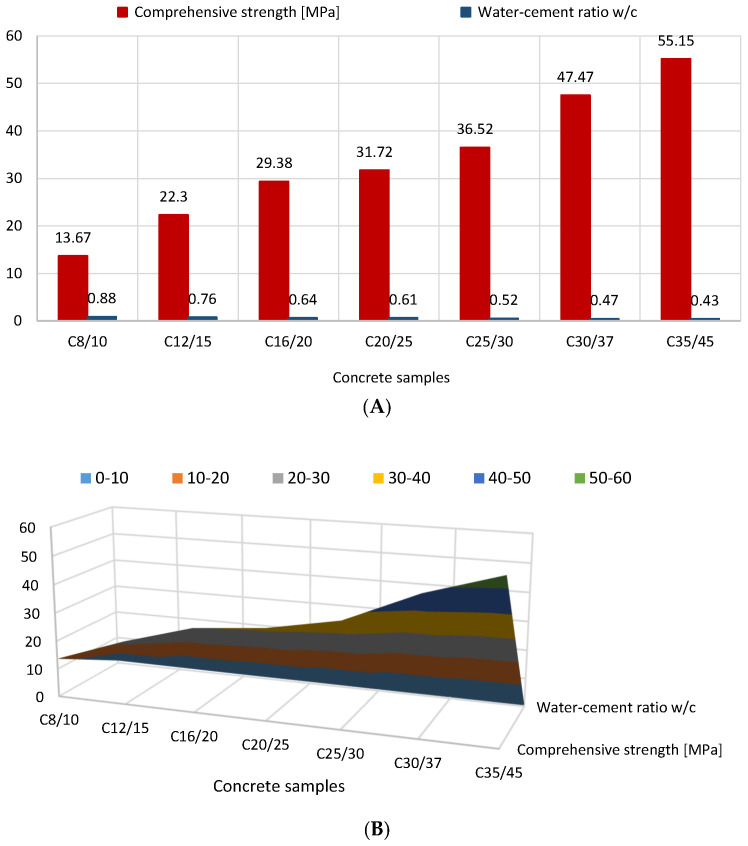
The relationship between the water-to-cement (w/c) ratio and the concrete compressive strength (version 2D: (**A**), 3D: (**B**)).

One of the most crucial factors affecting the strength of properly compacted concrete is the w/c ratio. When water is added to concrete mixtures to promote fluidity, the w/c value rises, and the strength of the concrete is compromised. As a result, it is advised to aim for low w/c ratios in order to produce concrete that is both strong and of high quality. The correlation between the water-to-cement ratio (w/c) and the compressive strength of concrete is depicted in Figure 8. It is clearly visible that mixtures with low water-to-cement ratios showed an increase in strength compared to mixtures with high water-to-cement ratios. In actual construction practice, mixes with a low water-to-cement ratios strengthen far more quickly than mixes with high water-to-cement ratios. The w/c ratio rises as the amount of water in the concrete mix grows; in other words, the more water that is in the concrete, the greater the w/c ratio, which has a negative effect on the strength of the concrete.

## 4. Conclusions

These investigations allowed us to show the relationship between the mechanical strength of waste-modified concretes and various parameters, including the composition of concrete mixes (amount of sand and gravel, cement, SSFA), water-to-cement (w/c) ratio, and sand point. Current Polish and European regulations allow the use of fly ash obtained from the combustion of sewage sludge as an additive for the preparation of concrete mixes [36,37]. Therefore, the addition of SSFA was used as a waste material resulting from circulating fluidized bed combustion (CFBC) technology. According to a review of the experimental studies, the following results were found:

1. The compressive strength of concrete was shown to increase as the class of concrete increased while keeping the appropriate composition of components (from 13.7 MPa (C8/10) to 55.2 MPa (C35/45)).
2. Concrete mixes with low w/c ratios and high cement contents showed increases in compressive strength compared to mixes with high w/c ratios and low cement contents.
3. The addition of SSFA as a by-product of municipal sewage sludge incineration did not reduce the compressive strength, so it can be successively used. There are no legal regulations regarding the requirements for the physical and chemical properties of SSFA from the combustion of municipal sewage sludge, and there are also no legal specifications regarding the possibility of their use in the production of concrete. Oxides of calcium, phosphorus, aluminum, silicon, and iron had the highest concentrations in SSFA samples. Utilizing the waste materials lowers the price of making concrete and prevents it from going to landfills.
4. According to these investigations, compressive strength increased when sand content decreased (from 990 kg to 620 kg) and sand point value decreased (from 0.54% to 0.32%). On the other hand, the compressive strength increased as the amount of 2/8 gravel (from 360 kg to 562) and 8/16 gravel (from 468 kg to 670 kg) increased.

The results of the experimentation revealed how specific components and parameters affected the compressive strength of concrete samples. The components of the concrete mixes and their mutual ratios should be selected in such a way that the execution of the concrete is as economical as possible while maintaining the minimal standards of quality, in particular, strength, durability, and consistency. The experimental outcomes supported the hypothesis that the thermal transformation of municipal sewage sludge into fly ash using CFBC technology improved the strength characteristics of concrete. The offered findings serve as the foundation for further study in this field. Further research on the influence of composition variability on the technical parameters of concretes containing fly ash from sewage sludge seems necessary, as does a correlation analysis between these variables and the strength properties of concrete samples.

## Figures and Tables

**Table 1:** Composition and properties of concrete mixes used in the tests.

Concrete Composition and Properties	Recipe Number of Mixes						
	510,471	515,471	520,470	525,410	530,412	537,412	545,460
	Concrete Class						
	C8/10	C12/15	C16/20	C20/25	C25/30	C30/37	C35/45
Sand 0/2 mm (kg)	990	810	730	695	690	620	620
Gravel 2/8 mm (kg)	360	455	521	497	525	555	562
Gravel 8/16 mm (kg)	468	560	645	610	640	670	642
CEM II/B-S 42.5 N (kg)	205	230	265	275	320	340	380
Water (kg)	180	174	170	169	166	160	163
Air content in the mixture (%)	2	1.9	1.9	1.9	1.9	2	4.5
Fly ash (kg)	90	80	75	70	65	60	55
Superplasticizer Sikament 400/30	—	—	2.12	3.03	3.52	3.4	4.56
Air-entraining admixture Sika^®^ Luftporenbildner LPS A-94	—	—	—	—	—	—	1.14
Sand point (%)	0.54	0.44	0.39	0.38	0.37	0.33	0.32
Water to cement ratio w/c	0.88	0.76	0.64	0.61	0.52	0.47	0.43

**Table 2:** The physico-chemical properties of 0/2 mm sand.

Compounds, Parameters	Content and Results
SiO_2_	>99.1%
Fe_2_O_3_	320 ppm
Al_2_O_3_	2600 ppm
CaO	240 ppm
MgO	70 ppm
Clay	0.2%
CaCO_3_	0.4%
Moisture	<0.1%
pH	7.0
Density	2.54 g/cm^3^

**Table 3:** The properties of 0/2 sand and 2/8 and 8/16 gravel.

Property	Sand, 0/2 mm	Gravel, 2/8 mm	Gravel, 8/16 mm
The content of mineral dust	0.65% category *f*_3_	0.46% category *f*_3_	0.42% category *f*_3_
The content of organic substances	Absence	Absence	Absence
Bulk density	1.71 kg/dm^3^	1.67 kg/dm^3^	1.78 kg/dm^3^
Flatness index	—	6.2%, category *FI*_10_	5.6%, category *FI*_10_
Absorptivity	—	0.58%	0.55%

**Table 4:** Chemical composition of CEM II/B-S 42.5 N cement.

Compound	CaO	SiO_2_	Al_2_O_3_	Fe_2_O_3_	MgO	Na_2_O	K_2_O	Na_2_O_eq_
Content (%)	53.41	28.38	6.76	2.54	4.26	0.45	0.62	0.74

**Table 5:** Granulometric composition of SSFA.

FA grain fractions (mm)	0–0.212	0.212–0.5	0.5–0.71	0.71–1.0	1.0–1.7	>1.7
Granulometric composition (%)	90.17 ± 1.2	8.64 ± 1.2	0.51 ± 0.07	0.68 ± 0.06	0	0

**Table 6:** Elemental composition of SSFA (SEM-EDS analysis).

Element	Ca	O	P	Al	Si	Fe	C	Mg	K	Zn	Ti	Na
Weight content (%)	7.3	46.8	6.8	12.9	13.2	3.0	1.59	2.34	4.35	0.46	0.35	0.85
Atomic content (%)	3.86	62.0	4.63	10.1	9.98	1.13	2.81	2.03	2.36	0.15	0.15	0.79
Compound	CaO	-	P_2_O_5_	Al_2_O_3_	SiO_2_	Fe_2_O_3_	CO_2_	MgO	K_2_O	ZnO	TiO_2_	Na_2_O
Content (%)	10.21	-	15.52	24.42	28.32	4.27	5.84	3.87	5.24	0.57	0.85	1.15

**Table 7:** Average strength test results of concrete mixes.

Class Concrete	Sample Weight (kg)	Density (kg/dm^3^)	Destructive Force (kN)	Compressive Strength (Mpa)
C8/10	7.385 ± 0.015	2.188 ± 0.005	307.83 ± 9.58	13.67 ± 0.41
C12/15	7.588 ± 0.023	2.248 ± 0.007	501.67 ± 85.53	22.3 ± 3.82
C16/20	7.771 ± 0.041	2.303 ± 0.012	661 ± 33.17	29.38 ± 1.46
C20/25	7.673 ± 0.053	2.274 ± 0.016	713.67 ± 15.99	31.72 ± 0.70
C25/30	7.655 ± 0.055	2.268 ± 0.016	821.5 ± 30.62	36.52 ± 1.39
C30/37	7.723 ± 0.051	2.288 ± 0.015	1068.3 ± 89.30	47.47 ± 3.95
C35/45	7.783 ± 0.047	2.306 ± 0.014	1240.83 ± 26.69	55.15 ± 1.18

## Data Availability

Data are contained within the article.

## Supplementary Materials

The following supporting information can be downloaded at: https://www.mdpi.com/article/10.3390/ma16114043/s1, Table S1: Strength test results of C8/10 class concrete; Table S2: Strength test results of C12/15 class concrete; Table S3: Strength test results of C16/20 class concrete; Table S4: Strength test results of C20/25 class concrete; Table S5: Strength test results of C25/30 class concrete; Table S6: Strength test results of C30/37 class concrete; Table S7: Strength test results of C35/45 class concrete.
