# Supplementary material for: Modification of Concrete Composition Doped by Sewage Sludge Fly Ash and Its Effect on Compressive Strength
Source: Materials (Basel). 2023 May 29;16(11):4043. doi: 10.3390/ma16114043 (PMC10254342; doi:10.3390/ma16114043)
Supplement: Supplementary file 1 [file materials-16-04043-s001.zip › materials-2305320-supplementary.pdf]

materials

MDPI

Supplementary Materials

Modification of Concrete Composition Doped by Sewage Sludge Fly Ash and Its Effect on Compressive Strength

Tomasz Kalak [1,*], Patrycja Szypura [1], Ryszard Cierpiszewski [1] and Małgorzata Ulewicz [2]

[1] Department of Industrial Products and Packaging Quality, Institute of Quality Science, Poznań University of Economics and Business, Niepodległości 10, 61-875 Poznań, Poland; ryszard.cierpiszewski@ue.poznan.pl (R.C.)
[2] Faculty of Civil Engineering, Częstochowa University of Technology, Akademicka 3 Street, 42-200 Częstochowa, Poland
* Correspondence: tomasz.kalak@ue.poznan.pl

Table S1. Strength test results of C8/10 class concrete.

S.No.	Sample weight [kg]	Density [kg/dm³]	Destructive force [kN]	Compressive strength [Mpa]
**1**	7.393	2.191	308	13.7
**2**	7.389	2.189	313	13.9
**3**	7.390	2.190	311	13.8
**4**	7.394	2.191	307	13.6
**5**	7.391	2.19	290	12.9
**6**	7.354	2.179	318	14.1
**Average**	**7.385**	**2.188**	**307.83**	**13.67**
**Standard deviation**	0.015	0.005	9.58	0.41

Table S2. Strength test results of C12/15 class concrete.

S.No.	Sample weight [kg]	Density [kg/dm³]	Destructive force [kN]	Compressive strength [Mpa]
**1**	7.601	2.252	502	22.3
**2**	7.605	2.253	620	27.6
**3**	7.560	2.240	589	26.2
**4**	7.556	2.239	412	18.3
**5**	7.603	2.253	452	20.1
**6**	7.603	2.253	435	19.3
**Average**	**7.588**	**2.248**	**501.67**	**22.3**
**Standard deviation**	0.023	0.007	85.53	3.82

Table S3. Strength test results of C16/20 class concrete.

S.No.	Sample weight [kg]	Density [kg/dm³]	Destructive force [kN]	Compressive strength [Mpa]
**1**	7.810	2.314	666	29.6
**2**	7.779	2.305	643	28.6
**3**	7.691	2.279	614	27.3
**4**	7.773	2.303	652	29.0

**5**	7.793	2.309	680	30.2
**6**	7.783	2.306	711	31.6
**Average**	**7.771**	**2.303**	**661**	**29.38**
**Standard deviation**	0.041	0.012	33.17	1.46

Table S4. Strength test results of C20/25 class concrete.

**S.No.**	**Sample weight [kg]**	**Density [kg/dm³]**	**Destructive force [kN]**	**Compressive strength [Mpa]**
**1**	7.712	2.285	711	31.6
**2**	7.707	2.284	709	31.5
**3**	7.680	2.276	727	32.3
**4**	7.726	2.289	713	31.7
**5**	7.602	2.252	688	30.6
**6**	7.614	2.256	734	32.6
**Average**	**7.673**	**2.274**	**713.67**	**31.72**
**Standard deviation**	0.053	0.016	15.99	0.70

Table S5. Strength test results of C25/30 class concrete.

**S.No.**	**Sample weight [kg]**	**Density [kg/dm³]**	**Destructive force [kN]**	**Compressive strength [Mpa]**
**1**	7.694	2.280	843	37.5
**2**	7.709	2.284	850	37.8
**3**	7.687	2.278	841	37.4
**4**	7.637	2.263	802	35.6
**5**	7.645	2.265	823	36.6
**6**	7.558	2.239	770	34.2
**Average**	**7.655**	**2.268**	**821.5**	**36.52**
**Standard deviation**	0.055	0.016	30.62	1.39

Table S6. Strength test results of C30/37 class concrete.

**S.No.**	**Sample weight [kg]**	**Density [kg/dm³]**	**Destructive force [kN]**	**Compressive strength [Mpa]**
**1**	7.701	2.282	999	44.4
**2**	7.758	2.299	1018	45.2
**3**	7.662	2.270	1148	51.0
**4**	7.708	2.284	960	42.7
**5**	7.705	2.283	1184	52.6
**6**	7.807	2.313	1101	48.9
**Average**	**7.723**	**2.288**	**1068.3**	**47.47**
**Standard deviation**	0.051	0.015	89.30	3.95

Table S7. Strength test results of C35/45 class concrete.

S.No.	Sample weight [kg]	Density [kg/dm³]	Destructive force [kN]	Compressive strength [Mpa]
**1**	7.792	2.309	1243	55.2
**2**	7.837	2.322	1276	56.7
**3**	7.790	2.308	1199	53.3
**4**	7.76	2.299	1224	54.4
**5**	7.817	2.316	1257	55.9
**6**	7.704	2.283	1246	55.4
**Average**	**7.783**	**2.306**	**1240.83**	**55.15**
**Standard deviation**	0.047	0.014	26.69	1.18
